# Supplementary material for: Pest control of aphids depends on landscape complexity and natural enemy interactions
Source: PeerJ. 2015 Jul 16;3:e1095. doi: 10.7717/peerj.1095 (PMC4699780; doi:10.7717/peerj.1095)
Supplement: Table S1 — Results without this factor are shown in Table 1 (see Methods §4 and Table 1 for details on the model selection procedure). Sets of 95% confidence models and weights of each predictor are given for all response variables. w, AIC weight compared to all possible models; w95%, AIC weight within the 95% model confidence set. Explanatory variables are A, initial number of aphids; M, management type of the nearest surrounding field (organic/conventional); L, landscape complexity (% seminatural habitat in the surrounding radius); D, sampling date (1–3); T, exclusion treatment (6 levels of natural enemy exclusion). [file peerj-03-1095-s001.docx]

**Table S1.** Effects of including initial aphid density on model results for aphid population growth, parasitism rate and syrphid fractions. Results without this factor are shown in Table 1 (see Methods §4 and Table 1 for details on the model selection procedure). Sets of 95% confidence models and weights of each predictor are given for all response variables. w: AIC weight compared to all possible models, w95%: AIC weight within the 95% model confidence set.

Explanatory variables are A: initial number of aphids; M: management type of the nearest surrounding field (organic / conventional); L: landscape complexity (% seminatural habitat in the surrounding radius); D: sampling date (1-3); T: exclusion treatment (6 levels of natural enemy exclusion)

Table S1 (continued)
