# Supplementary material for: Pest control of aphids depends on landscape complexity and natural enemy interactions
Source: PeerJ. 2015 Jul 16;3:e1095. doi: 10.7717/peerj.1095 (PMC4699780; doi:10.7717/peerj.1095)
Supplement: Table S2 — Sets of 95% confidence models and weights of each explanatory variable are shown for each response variable at scales from 100 to 1,000 m around fields. Lowest AIC values of the full model (not shown) and of the selected model (in bold) were obtained at the 700 m scale for aphid population growth, the 200 m scale for parasitism rates and the 900 m scale for syrphid fractions. w, AIC weight compared to all possible models; w95%, AIC weight within the 95% model confidence set. Explanatory variables are M, management type of the nearest surrounding field (organic/conventional); L, landscape complexity (% seminatural habitat in the surrounding radius); D, sampling date (1–3); T, exclusion treatment (6 levels of natural enemy exclusion). [file peerj-03-1095-s002.docx]

**Table S2.** Effect of scale on the response of A) average daily aphid population growth, B) parasitism rates, C) syrphid fractions. Sets of 95% confidence models and weights of each explanatory variable are shown for each response variable at scales from 100 to 1000 m around fields. Lowest AIC values of the full model (not shown) and of the selected model (in bold) were obtained at the 700 m scale for aphid population growth, the 200 m scale for parasitism rates and the 900 m scale for syrphid fractions. w: AIC weight compared to all possible models, w95%: AIC weight within the 95% model confidence set. Explanatory variables are M: management type of the nearest surrounding field (organic / conventional); L: landscape complexity (% seminatural habitat in the surrounding radius); D: sampling date (1-3); T: exclusion treatment (6 levels of natural enemy exclusion)

**A) Aphid population growth**

**B) Parasitism rates**

**B) Parasitism rates (continued)**

**C) Syrphid fractions**

**C) Syrphid fractions (continued)**
