# Supplementary material for: Pest control of aphids depends on landscape complexity and natural enemy interactions
Source: PeerJ. 2015 Jul 16;3:e1095. doi: 10.7717/peerj.1095 (PMC4699780; doi:10.7717/peerj.1095)
Supplement: Table S3 — This table shows the difference in slope between treatments, where slope is the effect of changes in % seminatural habitat around fields on response variables. Slopes are compared against zero (i.e., compared to no effect: estimates are then the absolute slope of the treatment across a gradient in % seminatural habitat) or are given relative to the slopes of other treatments. Treatments are O, open treatment without exclusion; -B, exclusion of birds; -G, exclusion of ground-dwellers; -G-B, exclusion of ground-dwellers and birds, but not flying insects; -F-B, exclusion of flying insects and birds; -G-F-B, control excluding all enemies but including herbivores. P-values are adjusted for the False Discovery Rate using the Benjamini–Hochberg correction. Significance codes are ‘***’ p < 0.001, ‘**’ p < 0.01, ‘*’ p < 0.05, ‘.’ p < 0.1. [file peerj-03-1095-s003.docx]

**Table S3.** Multiple comparisons of slopes for A) aphid population growth, B) parasitism rate, C) syrphid fraction measured at three sampling dates. This table shows the difference in slope between treatments, where slope is the effect of changes in % seminatural habitat around fields on response variables. Slopes are compared against zero (i.e. compared to no effect: estimates are then the absolute slope of the treatment across a gradient in % seminatural habitat) or are given relative to the slopes of other treatments. Treatments are *O*: open treatment without exclusion; *-B*: exclusion of birds; *-G*: exclusion of ground-dwellers; *-G-B*: exclusion of ground-dwellers and birds, but not flying insects; *-F-B*: exclusion of flying insects and birds; *-G-F-B*: control excluding all enemies but including herbivores. P-values are adjusted for the False Discovery Rate using the Benjamini-Hochberg correction. Significance codes are ‘***’ p<0.001, ‘**’ p<0.01, ‘*’ p<0.05, ‘.’ p<0.1.

| **A. Aphid population growth** | **Sampling date 1** | | |  |  |  |  | **Sampling date 2** | | |  |  |  |  | **Sampling date 3** | | |  |  |  | | | |
| --- | --- | --- | --- | --- | --- | --- | --- | --- | --- | --- | --- | --- | --- | --- | --- | --- | --- | --- | --- | --- | --- | --- | --- |
| Treatments | Estimate | SE | z value | p |  | p (adjusted) | | Estimate | SE | z value | p |  | p (adjusted) | | Estimate | SE | z value | p |  | p (adjusted) | | | |
| -G-F-B vs. zero | 0.009 | 0.002 | 3.61 | 0.000 | *** | 0.005 | ** | 0.006 | 0.001 | 4.84 | 0.000 | *** | 0.000 | *** | 0.001 | 0.001 | 1.16 | 0.245 |  | 0.783 |  | | |
| -G-F-B vs. -F-B | -0.002 | 0.003 | -0.67 | 0.505 |  | 0.673 |  | -0.002 | 0.001 | -1.65 | 0.099 | . | 0.192 |  | 0.001 | 0.001 | 0.66 | 0.509 |  | 0.937 |  | | |
| -G-F-B vs. -G-B | -0.002 | 0.003 | -0.71 | 0.477 |  | 0.673 |  | -0.004 | 0.001 | -2.64 | 0.008 | ** | 0.027 | * | 0.000 | 0.001 | -0.53 | 0.596 |  | 0.937 |  | | |
| -G-F-B vs. -G | -0.008 | 0.003 | -2.70 | 0.007 | ** | 0.028 | * | -0.004 | 0.001 | -3.07 | 0.002 | ** | 0.009 | ** | -0.001 | 0.001 | -0.75 | 0.455 |  | 0.937 |  | | |
| -G-F-B vs. -B | -0.008 | 0.003 | -2.70 | 0.007 | ** | 0.028 | * | -0.004 | 0.001 | -3.34 | 0.001 | *** | 0.004 | ** | -0.001 | 0.001 | -0.95 | 0.341 |  | 0.909 |  | | |
| -G-F-B vs. O | -0.008 | 0.003 | -2.73 | 0.006 | ** | 0.028 | * | -0.006 | 0.001 | -4.12 | 0.000 | *** | 0.000 | *** | -0.001 | 0.001 | -0.61 | 0.542 |  | 0.937 |  | | |
| -F-B vs. -G-B | 0.000 | 0.003 | -0.01 | 0.992 |  | 0.992 |  | 0.001 | 0.001 | 0.94 | 0.347 |  | 0.427 |  | 0.001 | 0.001 | 1.22 | 0.224 |  | 0.783 |  | | |
| -F-B vs. -G | 0.006 | 0.003 | 2.13 | 0.033 | * | 0.057 | . | 0.002 | 0.001 | 1.41 | 0.159 |  | 0.255 |  | 0.001 | 0.001 | 1.44 | 0.151 |  | 0.783 |  | | |
| -F-B vs. -B | 0.005 | 0.003 | 2.10 | 0.035 | * | 0.057 | . | 0.002 | 0.001 | 1.61 | 0.108 |  | 0.192 |  | 0.001 | 0.001 | 1.65 | 0.099 | . | 0.783 |  | | |
| -F-B vs. O | 0.006 | 0.003 | 2.15 | 0.032 | * | 0.057 | . | 0.003 | 0.001 | 2.47 | 0.014 | * | 0.036 | * | 0.001 | 0.001 | 1.30 | 0.194 |  | 0.783 |  | | |
| -G-B vs. -G | 0.006 | 0.003 | 2.38 | 0.017 | * | 0.039 | * | 0.001 | 0.001 | 0.51 | 0.612 |  | 0.653 |  | 0.000 | 0.001 | 0.23 | 0.822 |  | 0.937 |  | | |
| -G-B vs. -B | 0.005 | 0.002 | 2.41 | 0.016 | * | 0.039 | * | 0.001 | 0.001 | 0.67 | 0.505 |  | 0.577 |  | 0.000 | 0.001 | 0.43 | 0.668 |  | 0.937 |  | | |
| -G-B vs. O | 0.006 | 0.002 | 2.43 | 0.015 | * | 0.039 | * | 0.002 | 0.001 | 1.62 | 0.106 |  | 0.192 |  | 0.000 | 0.001 | 0.08 | 0.937 |  | 0.937 |  | | |
| -G vs. -B | -0.001 | 0.002 | -0.26 | 0.797 |  | 0.981 |  | 0.000 | 0.001 | 0.13 | 0.901 |  | 0.901 |  | 0.000 | 0.001 | 0.20 | 0.841 |  | 0.937 |  | | |
| -G vs. O | 0.000 | 0.002 | -0.10 | 0.920 |  | 0.981 |  | 0.001 | 0.001 | 1.08 | 0.280 |  | 0.406 |  | 0.000 | 0.001 | -0.15 | 0.883 |  | 0.937 |  | | |
| -B vs. O | 0.000 | 0.002 | 0.16 | 0.871 |  | 0.981 |  | 0.001 | 0.001 | 1.03 | 0.305 |  | 0.406 |  | 0.000 | 0.001 | -0.35 | 0.725 |  | 0.937 |  | | |
|  |  |  |  |  |  |  |  |  |  |  |  |  |  |  |  |  |  |  |  |  |  | | |
| **B. Parasitism rate** | **Sampling date 1** | | |  |  |  |  | **Sampling date 2** | | |  |  |  |  | **Sampling date 3** | | |  |  |  |  | | |
| Treatments | Estimate | SE | z value | p |  | p (adjusted) | | Estimate | SE | z value | p |  | p (adjusted) | | Estimate | SE | z value | p |  | p (adjusted) | | | |
| -G-F-B vs. zero | -0.006 | 0.118 | -0.05 | 0.960 |  | 0.967 |  | -0.080 | 0.115 | -0.70 | 0.486 |  | 0.778 |  | -0.168 | 0.105 | -1.60 | 0.111 |  | 0.352 | |  | |
| -F-B vs. zero | 0.251 | 0.132 | 1.90 | 0.057 | . | 0.340 |  | 0.222 | 0.128 | 1.74 | 0.082 | . | 0.437 |  | 0.247 | 0.131 | 1.89 | 0.059 | . | 0.317 | |  | |
| -G-B vs. zero | 0.356 | 0.132 | 2.70 | 0.007 | ** | 0.111 |  | 0.345 | 0.129 | 2.68 | 0.007 | ** | 0.119 |  | 0.381 | 0.132 | 2.88 | 0.004 | ** | 0.063 | | . | |
| -G vs. zero | 0.192 | 0.127 | 1.52 | 0.129 |  | 0.413 |  | 0.174 | 0.123 | 1.42 | 0.157 |  | 0.501 |  | 0.188 | 0.126 | 1.50 | 0.135 |  | 0.352 | |  | |
| -B vs. zero | 0.216 | 0.131 | 1.65 | 0.099 | . | 0.395 |  | 0.200 | 0.127 | 1.57 | 0.116 |  | 0.464 |  | 0.226 | 0.130 | 1.73 | 0.083 | . | 0.333 | |  | |
| O vs. zero | 0.221 | 0.119 | 1.85 | 0.064 | . | 0.340 |  | 0.207 | 0.116 | 1.78 | 0.074 | . | 0.437 |  | 0.236 | 0.119 | 1.99 | 0.047 | * | 0.317 | |  | |
| -F-B vs. -G-B | -0.105 | 0.140 | -0.75 | 0.455 |  | 0.809 |  | -0.123 | 0.136 | -0.91 | 0.364 |  | 0.646 |  | -0.134 | 0.139 | -0.96 | 0.335 |  | 0.536 | |  | |
| -F-B vs. -G | 0.059 | 0.134 | 0.44 | 0.658 |  | 0.967 |  | 0.048 | 0.130 | 0.37 | 0.713 |  | 0.951 |  | 0.059 | 0.133 | 0.44 | 0.657 |  | 0.920 | |  | |
| -F-B vs. -B | 0.036 | 0.138 | 0.26 | 0.796 |  | 0.967 |  | 0.022 | 0.134 | 0.17 | 0.869 |  | 0.951 |  | 0.021 | 0.137 | 0.16 | 0.875 |  | 0.934 | |  | |
| -F-B vs. O | 0.030 | 0.130 | 0.23 | 0.815 |  | 0.967 |  | 0.015 | 0.126 | 0.12 | 0.907 |  | 0.951 |  | 0.011 | 0.129 | 0.09 | 0.931 |  | 0.934 | |  | |
| -G-B vs. -G | 0.164 | 0.136 | 1.21 | 0.228 |  | 0.608 |  | 0.171 | 0.133 | 1.29 | 0.197 |  | 0.525 |  | 0.193 | 0.136 | 1.43 | 0.154 |  | 0.352 | |  | |
| -G-B vs. -B | 0.140 | 0.139 | 1.01 | 0.313 |  | 0.627 |  | 0.145 | 0.136 | 1.07 | 0.284 |  | 0.568 |  | 0.156 | 0.139 | 1.12 | 0.262 |  | 0.468 | |  | |
| -G-B vs. O | 0.135 | 0.130 | 1.04 | 0.299 |  | 0.627 |  | 0.138 | 0.127 | 1.09 | 0.278 |  | 0.568 |  | 0.145 | 0.130 | 1.12 | 0.263 |  | 0.468 | |  | |
| -G vs. -B | -0.024 | 0.129 | -0.19 | 0.854 |  | 0.967 |  | -0.026 | 0.125 | -0.21 | 0.836 |  | 0.951 |  | -0.038 | 0.128 | -0.30 | 0.768 |  | 0.934 | |  | |
| -G vs. O | -0.029 | 0.121 | -0.24 | 0.812 |  | 0.967 |  | -0.033 | 0.118 | -0.28 | 0.778 |  | 0.951 |  | -0.048 | 0.121 | -0.40 | 0.690 |  | 0.920 | |  | |
| -B vs. O | -0.005 | 0.125 | -0.04 | 0.967 |  | 0.967 |  | -0.007 | 0.122 | -0.06 | 0.951 |  | 0.951 |  | -0.010 | 0.124 | -0.08 | 0.934 |  | 0.934 | |  | |
|  |  |  |  |  |  |  |  |  |  |  |  |  |  |  |  |  |  |  |  |  | |  | |
|  |  |  |  |  |  |  |  |  |  |  |  |  |  |  |  |  |  |  |  |  | |  | |
| **C. Syrphid fraction** | **Sampling date 1** | | |  |  |  |  | **Sampling date 2** | | |  |  |  |  | **Sampling date 3** | | |  |  |  | |  | |
| Treatments | Estimate | SE | z value | p |  | p (adjusted) | | Estimate | SE | z value | p |  | p (adjusted) | | Estimate | SE | z value | p |  | p (adjusted) | | | |
| -G-F-B vs. zero | -0.116 | 0.106 | -1.09 | 0.274 |  | 0.487 |  | -0.199 | 0.103 | -1.93 | 0.054 | . | 0.172 |  | -0.170 | 0.093 | -1.84 | 0.067 | . | 0.226 | | |  |
| -F-B vs. zero | 0.207 | 0.107 | 1.93 | 0.054 | . | 0.215 |  | 0.208 | 0.106 | 1.96 | 0.050 | * | 0.172 |  | 0.192 | 0.106 | 1.81 | 0.071 | . | 0.226 | | |  |
| -G-B vs. zero | 0.366 | 0.116 | 3.14 | 0.002 | ** | 0.027 | * | 0.359 | 0.114 | 3.14 | 0.002 | ** | 0.027 | * | 0.355 | 0.116 | 3.07 | 0.002 | ** | 0.035 | | | * |
| -G vs. zero | 0.181 | 0.106 | 1.70 | 0.089 | . | 0.253 |  | 0.172 | 0.104 | 1.65 | 0.099 | . | 0.226 |  | 0.178 | 0.106 | 1.68 | 0.093 | . | 0.248 | | |  |
| -B vs. zero | 0.292 | 0.114 | 2.55 | 0.011 | * | 0.083 | . | 0.290 | 0.114 | 2.55 | 0.011 | * | 0.086 | . | 0.292 | 0.114 | 2.56 | 0.011 | * | 0.085 | | | . |
| O vs. zero | 0.249 | 0.103 | 2.42 | 0.016 | * | 0.083 | . | 0.237 | 0.101 | 2.34 | 0.019 | * | 0.103 |  | 0.244 | 0.103 | 2.38 | 0.017 | * | 0.093 | | | . |
| -F-B vs. -G-B | -0.159 | 0.110 | -1.45 | 0.147 |  | 0.337 |  | -0.151 | 0.108 | -1.40 | 0.161 |  | 0.321 |  | -0.163 | 0.109 | -1.49 | 0.136 |  | 0.272 | | |  |
| -F-B vs. -G | 0.026 | 0.106 | 0.25 | 0.806 |  | 0.806 |  | 0.036 | 0.105 | 0.34 | 0.735 |  | 0.777 |  | 0.014 | 0.106 | 0.13 | 0.894 |  | 0.894 | | |  |
| -F-B vs. -B | -0.085 | 0.111 | -0.77 | 0.443 |  | 0.631 |  | -0.082 | 0.110 | -0.74 | 0.458 |  | 0.655 |  | -0.100 | 0.111 | -0.90 | 0.368 |  | 0.535 | | |  |
| -F-B vs. O | -0.042 | 0.103 | -0.41 | 0.682 |  | 0.730 |  | -0.029 | 0.101 | -0.28 | 0.777 |  | 0.777 |  | -0.053 | 0.102 | -0.51 | 0.608 |  | 0.695 | | |  |
| -G-B vs. -G | 0.185 | 0.111 | 1.67 | 0.095 | . | 0.253 |  | 0.187 | 0.109 | 1.72 | 0.086 | . | 0.226 |  | 0.177 | 0.110 | 1.60 | 0.109 |  | 0.249 | | |  |
| -G-B vs. -B | 0.074 | 0.113 | 0.66 | 0.512 |  | 0.631 |  | 0.070 | 0.111 | 0.62 | 0.532 |  | 0.655 |  | 0.063 | 0.113 | 0.56 | 0.575 |  | 0.695 | | |  |
| -G-B vs. O | 0.117 | 0.106 | 1.10 | 0.271 |  | 0.487 |  | 0.123 | 0.104 | 1.18 | 0.239 |  | 0.425 |  | 0.110 | 0.106 | 1.04 | 0.298 |  | 0.482 | | |  |
| -G vs. -B | -0.111 | 0.110 | -1.01 | 0.313 |  | 0.501 |  | -0.117 | 0.109 | -1.07 | 0.284 |  | 0.455 |  | -0.114 | 0.110 | -1.03 | 0.301 |  | 0.482 | | |  |
| -G vs. O | -0.068 | 0.102 | -0.67 | 0.503 |  | 0.631 |  | -0.064 | 0.100 | -0.64 | 0.521 |  | 0.655 |  | -0.067 | 0.102 | -0.66 | 0.512 |  | 0.683 | | |  |
| -B vs. O | 0.043 | 0.106 | 0.41 | 0.684 |  | 0.730 |  | 0.053 | 0.105 | 0.51 | 0.613 |  | 0.701 |  | 0.047 | 0.106 | 0.45 | 0.656 |  | 0.700 | | |  |
