# Supplementary figures and images for: Pest control of aphids depends on landscape complexity and natural enemy interactions
Source: PeerJ. 2015 Jul 16;3:e1095. doi: 10.7717/peerj.1095 (PMC4699780; doi:10.7717/peerj.1095)

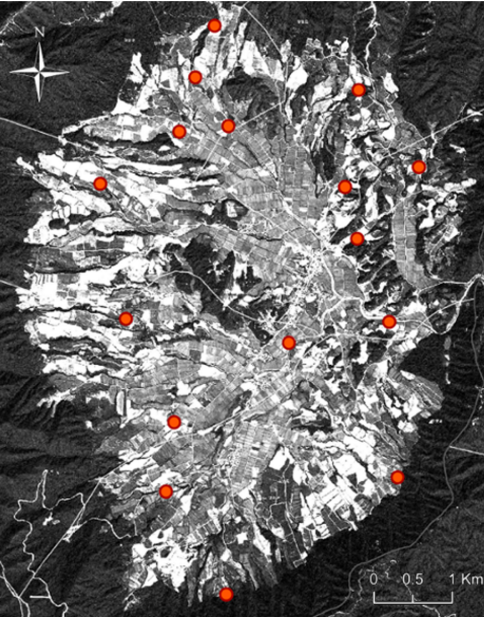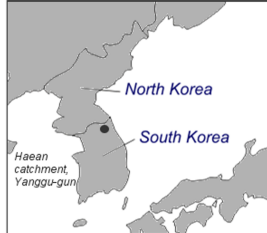

Supplement: Figure S1 — Location of the Haean agricultural landscape (South Korea) and of 16 experimental cabbage plots (red dots; 2 plots outside the catchment are not shown). Mean distance between fields was 3.2 ± 0.1 km (mean ± SE). Minimum distance was 211 m. See Statistics (Methods) for details on accounting for site proximity in models. Satellite image modified from Cnes/Spot Image (Google Maps ©2013). [file peerj-03-1095-s004.pdf]

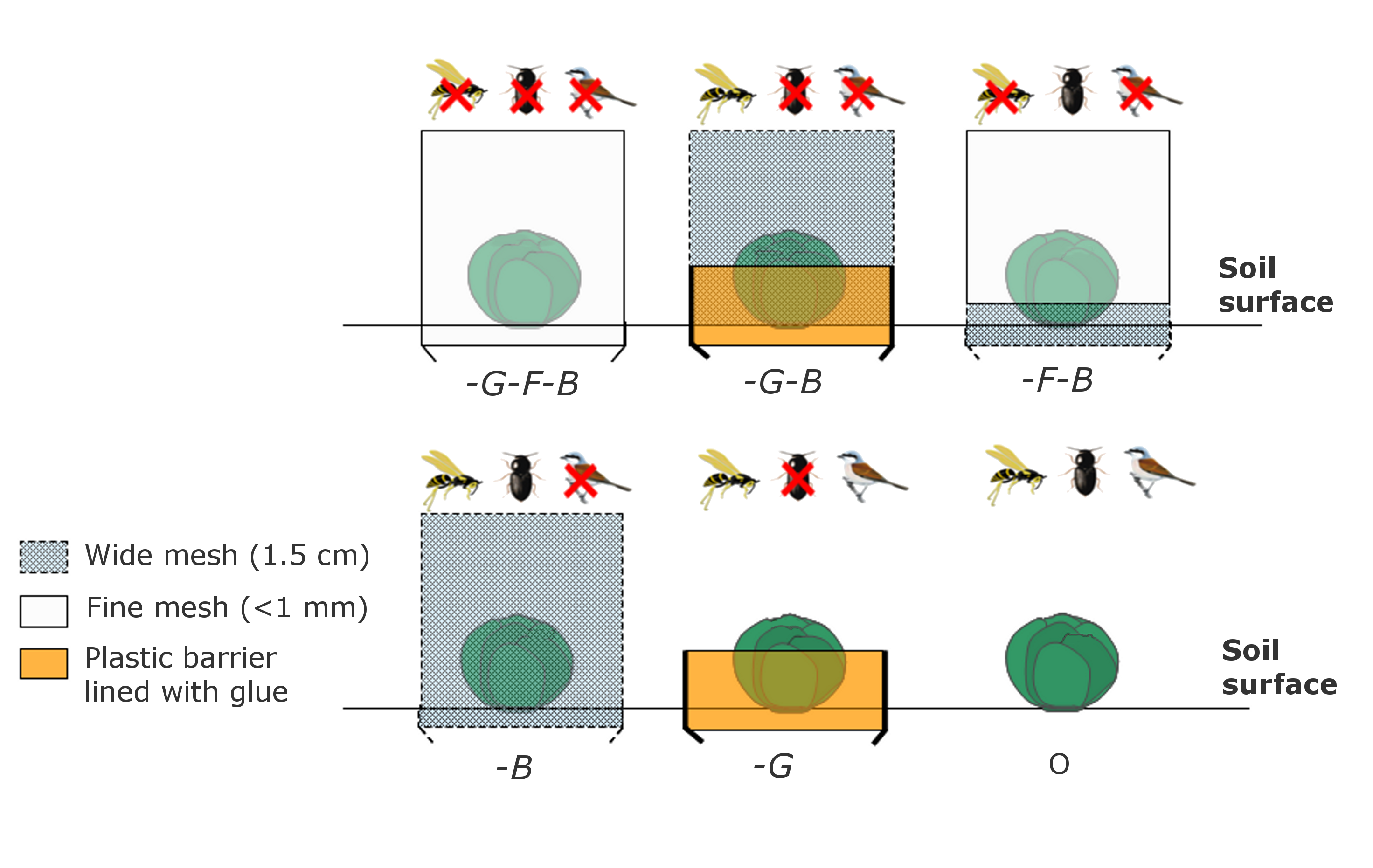

Supplement: Figure S2 — Exclosures are shown in a lateral view. Each exclosure contained four cabbages and was dug 10-20 cm into the ground. The same number of aphids was deposited on all plants of a given plot. Treatments are: O, open treatment, no exclusion; -B, exclusion of birds; -G, exclusion of ground-dwellers; -F-B, exclusion of flying insects and birds; -G-B, exclusion of ground-dwellers and birds, but not flying insects; -G-B-F, control; exclusion of all enemies. [file peerj-03-1095-s005.png]

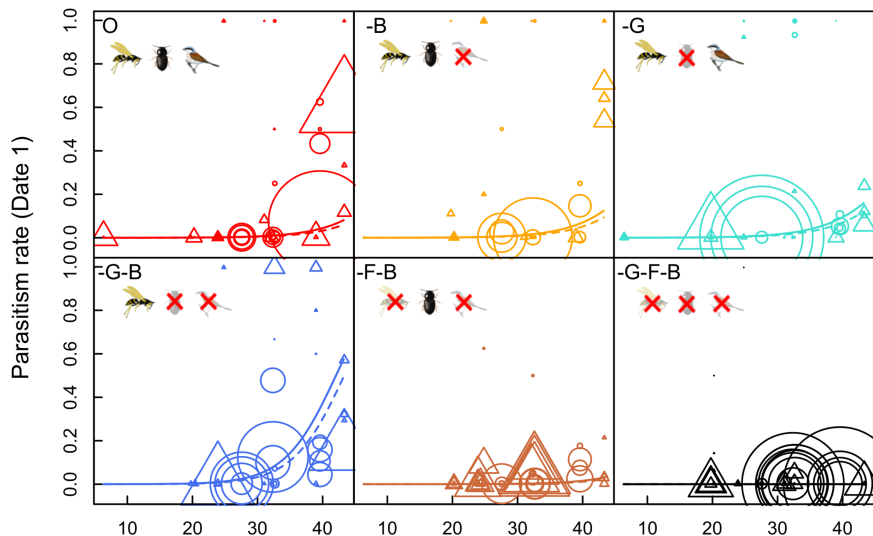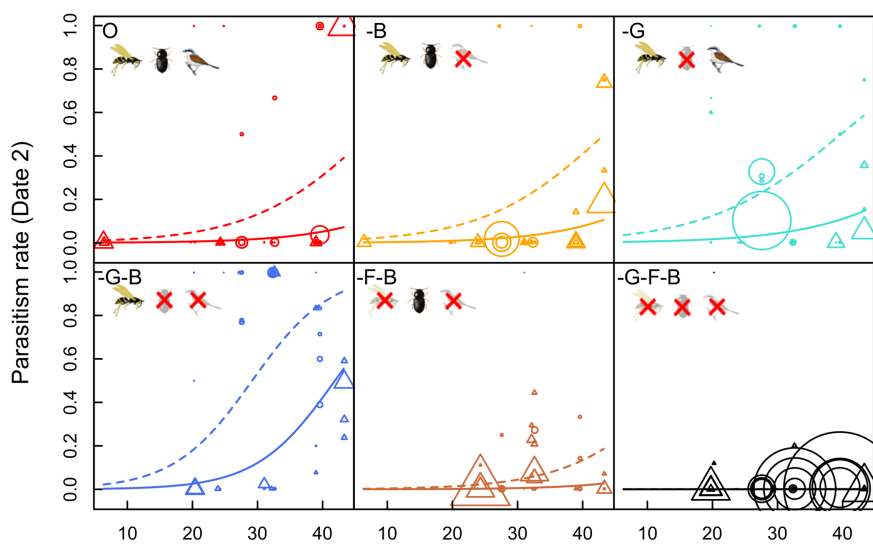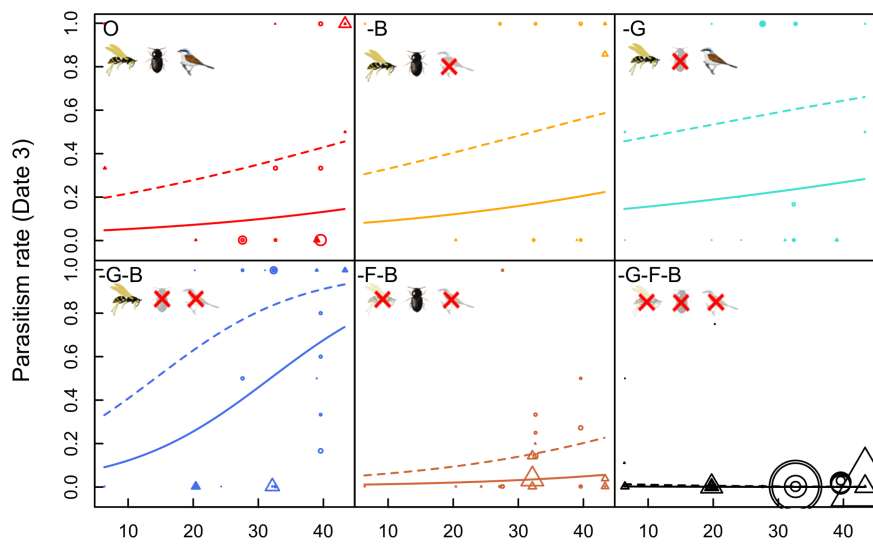

Percent seminatural habitat

Supplement: Figure S4 — Data points are given for each date and treatment. The area of each symbol is proportional to the total number of (parasitized + non-parasitized) aphids in the corresponding rate. Regression lines represent predictions of binomial GLMMs. Landscape complexity is defined as % seminatural habitat in a 200 m radius around plots (the most predictive scale of analysis for this response). Triangles and solid lines: organic management of the nearest surrounding field (13 plots), circles and dashed lines: conventional management of the nearest surrounding field (5 plots). O, open treatment without exclusion; -G, exclusion of ground-dwellers; -B, exclusion of birds; -F-B, exclusion of flying insects and birds; -G-B, exclusion of ground-dwellers and birds, but not flying insects; -G-F-B, control excluding all enemies but including herbivores. [file peerj-03-1095-s007.pdf]

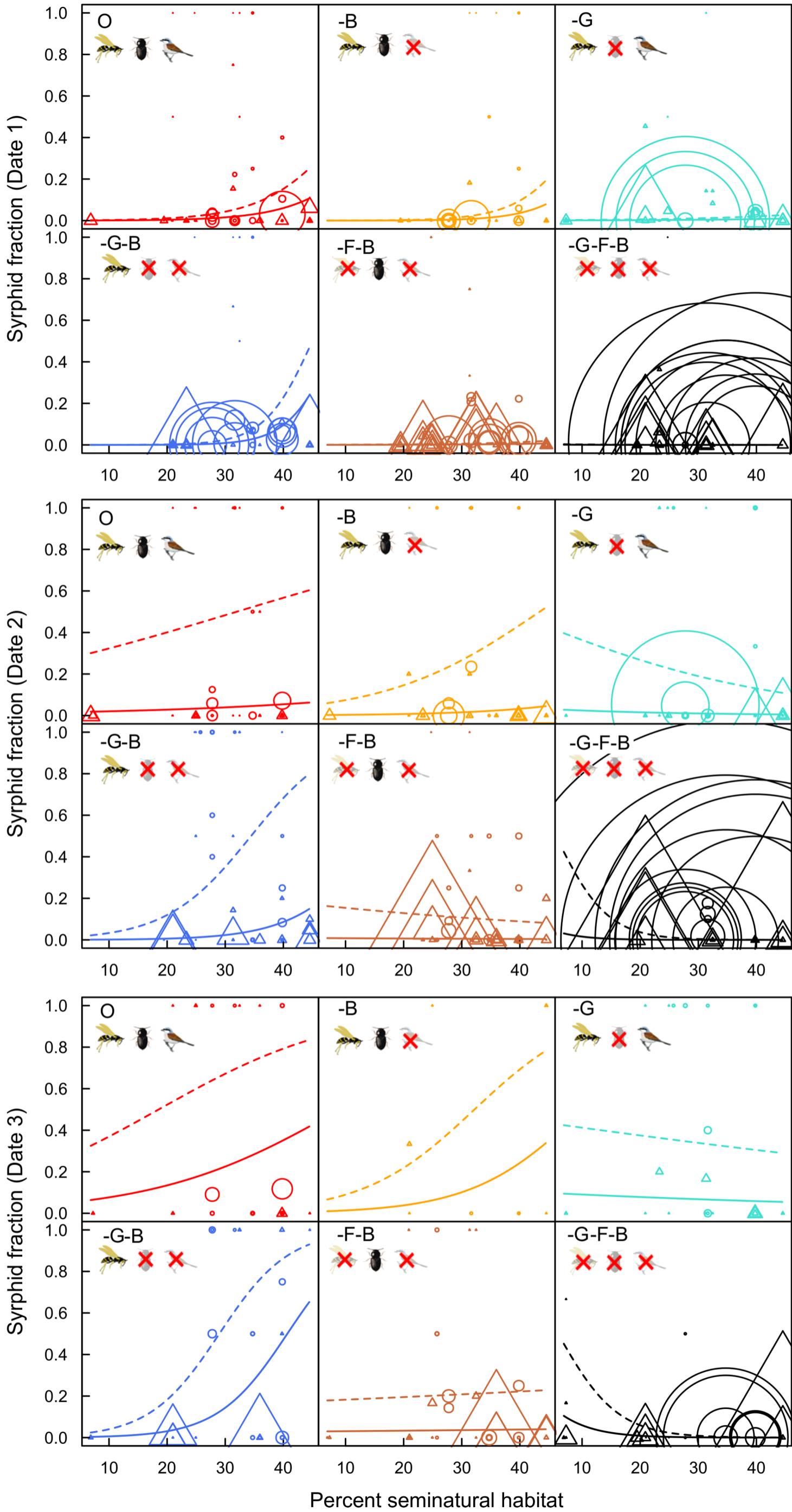

Supplement: Figure S5 — Data points are given for each date and treatment. The area of each symbol is proportional to the total number of syrphids + aphids in the corresponding fraction. Regression lines represent predictions of binomial GLMMs. Landscape complexity is defined as % seminatural habitat in a 900 m radius around plots (the most predictive scale of analysis for this response). Triangles and solid lines: organic management of the nearest surrounding field (13 plots), circles and dashed lines: conventional management of the nearest surrounding field (5 plots). O, open treatment without exclusion; -G, exclusion of ground-dwellers; -B, exclusion of birds; -F–B, exclusion of flying insects and birds; -G-B, exclusion of ground-dwellers and birds, but not flying insects; -G–F–B, control excluding all enemies but including herbivores. [file peerj-03-1095-s008.pdf]
