# Supplementary material for: Pest control of aphids depends on landscape complexity and natural enemy interactions
Source: PeerJ. 2015 Jul 16;3:e1095. doi: 10.7717/peerj.1095 (PMC4699780; doi:10.7717/peerj.1095)
Supplement: Figure S3 — Data points are given for each round and treatment. Each point represents one plant (i.e., sampling point). Four plants were sampled per exclusion treatment and landscape. Non-independence of sampling points within treatments and landscapes is accounted for in model random effects (Methods). Regression lines represent model-averaged predictions (Methods). Landscape complexity is defined as % seminatural habitat in a 700 m radius around plots (results at other scales are shown in Table S1). Full points and solid lines: organic management of the nearest surrounding field (13 plots), open points and dashed lines: conventional management of the nearest surrounding field (5 plots). O, open treatment without exclusion; -G, exclusion of ground-dwellers; -B, exclusion of birds; -F-B, exclusion of flying insects and birds; -G-B, exclusion of ground-dwellers and birds, but not flying insects; -G-F-B, control excluding all enemies but including herbivores. [file peerj-03-1095-s006.pdf]

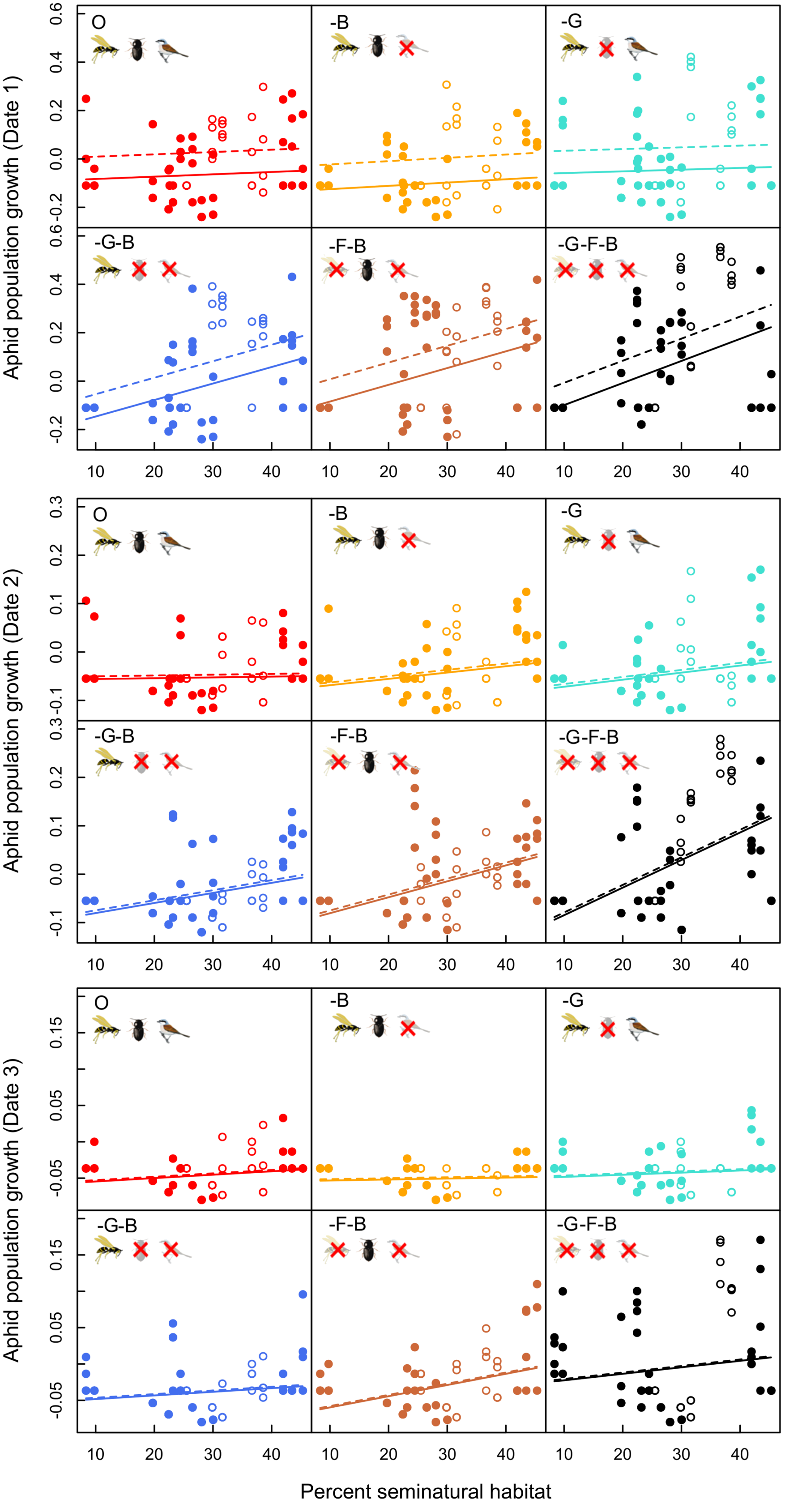

Aphid population growth (Date 1)
Aphid population growth (Date 2)
Aphid population growth (Date 3)
O
-B
-G
-G-B
-F-B
-G-F-B
Percent seminatural habitat
